# RHB-104 triple antibiotics combination in culture is bactericidal and should be effective for treatment of Crohn’s disease associated with *Mycobacterium paratuberculosis*

**DOI:** 10.1186/s13099-016-0115-3

**Published:** 2016-06-14

**Authors:** Karel P. Alcedo, Saisathya Thanigachalam, Saleh A. Naser

**Affiliations:** Burnett School of Biomedical Sciences, College of Medicine, University of Central Florida, Orlando, FL USA

**Keywords:** *Mycobacterium paratuberculosis*, Crohn’s disease, RHB-104, MIC, Antibiotics, IBD

## Abstract

**Background:**

*Mycobacterium avium* subspecies *paratuberculosis* (MAP) has been implicated as an etiological agent of Crohn’s disease (CD), a debilitating chronic inflammatory bowel disease. Clarithromycin (CLA), clofazimine (CLO), rifabutin (RIF) and other antibiotics have been used individually or in combinations with other drugs to treat mycobacterial diseases including CD. The treatment has varied by regimen, dosage, and duration, resulting in conflicting outcomes and additional suffering to the patients. RHB-104, a drug formula with active ingredients composed of (63.3 %) CLA, (6.7 %) CLO, and (30 %) RIF, has been recently subjected to investigation in an FDA approved Phase III clinical trial to treat patients with moderate to severe CD. In this study, we determined the efficacy of RHB-104 active ingredients against MAP strains isolated from the blood, tissue, and milk of CD patients. Based on fluorescence quenching technology using the Bactec MGIT Para-TB medium, we determined the minimum inhibitory concentration (MIC) of CLA, CLO, RIF individually and in dual and triple combinations against 16 MAP clinical strains and 19 other mycobacteria.

**Results:**

The MIC of all drugs against 35 different mycobacteria ranged between 0.25–20 μg/mL. However, the MIC of RHB-104 active ingredients regimen was the lowest at 0.25–10 μg/mL compared to the MIC of the other drugs at 0.5–20 μg/mL. The components of RHB-104 active ingredients at their individual concentrations or in dual combinations were not effective against all microorganisms compared to the triple combinations at MIC level. The MIC of CLA–CLO, CLA–RIF, and CLO–RIF regimens ranged between 0.5–1.25 μg/mL compared to 0.25 μg/mL of bactericidal effect of the triple combination.

**Conclusion:**

The data clearly demonstrated that lower concentrations of the triple combination of RHB-104 active ingredients provided synergistic anti-MAP growth activity compared to individual or dual combinations of the drugs. Consequently, this is favorable and should lead to tolerable dosage that is desirable for long-term treatment of CD and *Mycobacterium avium* complex disease.

## Background

Crohn’s disease (CD), a chronic inflammatory bowel disease, is caused by the interplay between genetic predisposition, immune dysregulation, and exposure to environmental factors. CD affects about 10.7 per 100,000 people-years in North America [1], and 6.3 per 100,000 people-years in Europe [2]. Despite the low incidence in Asia [3] and Africa, recent epidemiological studies have shown an increasing incidence of affected individuals in these continents [4–6]. Patients diagnosed with CD suffer from excessive and nocturnal diarrhea, abdominal pain, and rapid weight loss, all of which affect their quality of life [7]. A cobblestoned appearance of the mucosal layer and granulomas scattered in the distal ileum and colon is observed in CD patients [8]. These clinical and pathological manifestations have been observed in Johne’s disease, a chronic granulomatous inflammation of the intestines in ruminants [9]. Johne’s disease is caused by an intracellular pathogen called *Mycobacterium avium* subspecies *paratuberculosis* (MAP), a member of the *M. avium* complex (MAC) [9]. Our research group has cultured and detected MAP from the tissue, milk and blood samples from CD patients, showing zoonosis.

Medical treatment of CD includes anti-inflammatory drugs, immunosuppressants, nutritional therapy, and antibiotics. Anti-inflammatory drugs and immunosuppresants have been known to alleviate symptoms in CD; however, these commonly used medications have also been shown to have anti-MAP activity, particularly bacteriostatic effects [10]. Monoclonal antibodies such as Infliximab (Remicade) and Adalimumab (Humira) decrease the pro-inflammatory mediators and cytokines, which manages the symptoms experienced by CD patients, but relapse occurs after cessation of treatment [11–13]. These treatments present with documented significant adverse effects that include but are not limited to dependency on steroids, hypersensitivity, and potential excessive immune suppression leading to susceptibility to pathogens [14, 15]. They also do not address the possible inhibition of MAP as a zoonotic agent in CD, leading to inadequate treatment. Most often, patients require surgical intervention, which includes laparoscopy, strictureplasty, anastomosis, or bypass surgery [16, 17]. These surgical procedures are costly and time-consuming, and they alter patients’ lifestyles, especially when there is a recurrence of CD.

**Fig. 1:**
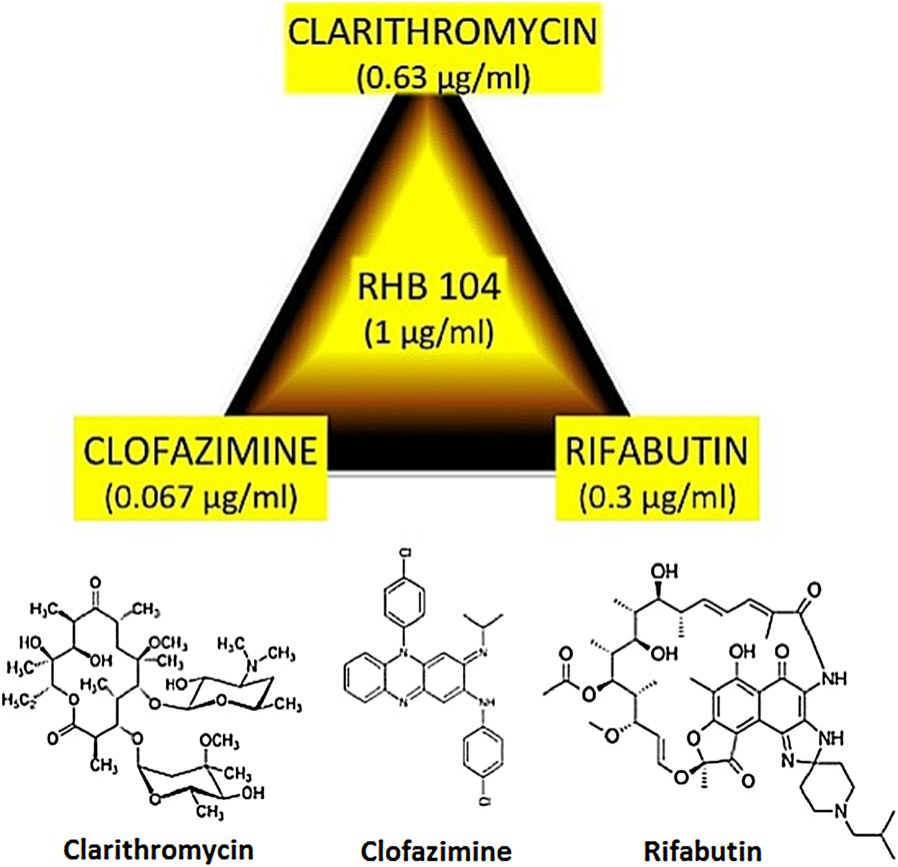
RHB-104 formula

Randomized clinical trials using antibiotic drugs called anti-MAP regimen in CD patients have shown promising results [18, 19]. Several studies reported that CD patients on rifabutin (RIF) and clarithromycin (CLA) regimen achieved complete healing of ulcers seen after >6 months of treatment. A 2007 case study reported that a patient, who was suffering from the recurrence of severe CD and was being treated with anti-inflammatory drugs, attained complete clinical remission using anti-MAP therapy [20]. As shown in Fig. 1, a pill of RHB-104 (RedHill Biopharma) active ingredients contains 95 mg CLA (0.63 %), 10 mg CLO (0.067 %), and 45 mg RIF (0.30 %). The chemical design of RHB-104 formula possesses significant potential advantages in drug administration and patient compliance. This study is designed to evaluate the active ingredients of RHB-104 in vitro against clinical MAP strains from CD patients. An effective anti-MAP therapy for CD is vital for clinical evaluation of MAP association with CD. Elimination of MAP concurrent with healing may result with cure in CD patients.

## Methods

### Mycobacterial strains and growth conditions

A total of 35 *Mycobacterium* species were used in this study (Table 1). It includes 16 clinical MAP strains, which were isolated from different types of clinical specimens from CD patients, 10 *Mycobacterium avium* strains, and 9 other *Mycobacterium* species. Mycobacteria were cultured in BD Bactec™ MGIT™ Para-TB medium (Sparks, MD) with growth supplement. Mycobactin J was added to culture media inoculated with MAP [21]. They were incubated at 37 °C and growth was measured initially using the UV illuminator (Andromeda). The culture medium contains a fluorescent molecule embedded in silicone that is sensitive to oxygen and will fluoresce in the presence of active respiring mycobacteria. Fluorescence quenching or the absence of fluorescence is indicative of no growth. Mycobacterial growth was quantified following incubation in a BD Bactec™ MGIT™ 320 Analyzer, which measures the intensity of emitted fluorescence and converts the value to a measurable growth unit. A growth unit of 75 or higher is indicative of bacterial growth. The culture media were incubated at 37 °C for a total of 6 months.

**Table 1:** *In*-*vitro* activity of RHB-104 and its individual active ingredients against clinical Mycobacterium strains

Microorganism	Minimum inhibitory concentration (μg/mL)			
	CLA	CLO	RIF	CLA–CLO–RIF
Clinical MAP strains				
MAP UCF 3	0.25	1	0.5	0.25
MAP UCF 4	0.25	0.5	0.5	0.25
MAP UCF 5	0.25	0.5	0.5	0.25
MAP UCF 7	1	0.5	0.5	0.25
MAP UCF 8	1	0.5	0.5	0.25
MAP UCF 10	10	>10	>10	10
MAP Strain 1	1	1	1	0.25
MAP Strain 3	1	1	1	0.25
MAP Strain 7	1	1	1	0.25
MAP Strain 8B	1	1	1	0.25
MAP MS 137	4	4	>6	4
MAP MS 185	4	6	>6	4
MAP Para 18	1	>1	>1	0.25
MAP Ben	1	>1	1	0.25
MAP Kay	0.5	1	2	0.25
MAP Linda	0.5	2	0.5	0.25
Non-MAP strains				
*M. avium*	4	>6	>6	4
*M. avium* NEZ	>1	>1	>1	>1
*M. avium* JF1	>20	>20	>20	>20
*M. avium* JF2	>6	>6	>6	>4
*M. avium* JF3	>6	>6	>6	>4
*M. avium* JF4	>6	>6	>6	>4
*M. avium* JF5	>6	>6	>6	4
*M. avium* JF6	>6	>6	>6	4
*M. avium* JF 7	>6	>6	>6	>4
*M. avium* JF8	>6	>6	>6	4
*M. intracellulare* LM1-A	>6	>6	>6	4
*M. smegmatis*	>6	>6	>6	6
*M. chelonae*	>10	10	>10	>10
*M. fortuitum*	>10	>10	>10	>10
*M. scrofulaceum*	10	10	10	10
*M. terrae*	10	10	10	10
*M. tuberculosis*	10	10	10	10
*M. xenopi*	10	10	10	10
*M. vallae*	10	10	10	10

*CLA* clarithromycin, *CLO* clofazimine, *RIF* rifabutin, *CLA–CLO–RIF* mimics RHB-104 active ingredients solution

### DNA extraction and IS900 nested PCR analysis

All mycobacterial cultures were subjected to *IS900* nested PCR analysis to confirm their DNA identity. DNA extraction was followed per protocol as published by our group [22]. Nested PCR was performed using P90/P91 primers (5′-GTTCGGGGCCGTCGCTTAGG-3′/5′-GAGGTCGATCGCCCACGTGA-3′) to amplify a 398 bp of the *IS900* gene in MAP genome. The second primers for Nested PCR were AV1/AV2 (5′-ATGTGGTTGCTGTGTTGGATGG-3′/5′-CCGCCGCAATCAACTCCAG-3′) to amplify a 298 bp from the initial products. Amplified PCR products were analyzed on a 2 % agarose gel [22].

### Antibiotic drug susceptibility testing

CLA, CLO, RIF, were kindly provided by RedHill Biopharma. The stock solution for CLA at 1 mg/mL was prepared using sodium acetate in water (pH 5.0). The stock solution for CLO at 1 mg/mL was prepared using hydrochloric acid and sodium dodecyl sulfate in water. The stock solution for RIF at 1 mg/mL was prepared using absolute methanol [23]. RHB-104 active ingredients solution was prepared at a final concentration of 1 mg/mL by combining the 3 individual dissolved drugs at their respective percent composition in RHB-104 (63.3 % CLA, 30 % RIF, and 6.7 % CLO; Fig. 1).

The minimum inhibitory concentration (MIC) for each drug against mycobacteria was determined by the lowest amount of drug tested that completely inhibited bacterial growth indicated by absence of fluorescence from the culture tubes, and/or a growth index of zero. All mycobacterial strains were inoculated in BD Bactec™ MGIT™ Para-TB medium tube with 10^5^–10^6^ colony-forming unit in the presence of the drugs at concentrations ranging between 0.1–20 μg/mL. Controls were established in culture medium without any antibiotic drugs. All cultures were incubated at 37 °C and read daily for growth for up to 6 months.

### Synergistic effects analysis

Initially, to determine any synergistic effects of RHB-104 active ingredients, CLA, CLO, and RIF at their respective concentrations in MIC levels of RHB-104 were tested individually against mycobacterial strains and were compared to CLA–CLO–RIF solutions (Table 2). Bacterial resistance or susceptibility to these very low concentrations of CLA, CLO, and RIF was determined. In addition, regimens of 2-drug combinations CLA-CLO, CLA-RIF, CLO-RIF were also compared to CLA-CLO-RIF solutions. These combinations were prepared at concentrations between 0.1 and 1.0 μg/mL of RHB-104 and used against MAP strains cultured in BD Bactec™ MGIT™ TB Medium. Growth supplements and culture conditions were similar to those in earlier experiments. Percent inhibition of these dual regimens was determined by calculating bacterial growth index at mid-logarithmic phase in culture with the drugs compared to the control without any drug.

**Table 2:** Susceptibility of mycobacterial strains to CLA, CLO, and RIF at their concentrations in the MIC of CLA–CLO–RIF (RHB-104 active ingredients)

Organism	Source	MIC	Comparison analysis					
		CLA–CLO–RIF	CLA (63.3 %)		CLO (6.7 %)		RIF (30 %)	
		μg/mL	μg/mL	Susceptibility	μg/mL	Susceptibility	μg/mL	Susceptibility
Clinical MAP strains								
MAP UCF 3	Tissue	0.25	0.158	R	0.017	R	0.075	R
MAP UCF 4	Tissue	0.25	0.633	R	0.067	R	0.3	R
MAP UCF 5	Tissue	0.25	0.633	R	0.067	R	0.3	R
MAP UCF 7	Tissue	0.25	0.633	R	0.067	R	0.3	R
MAP UCF 8	Tissue	0.25	0.633	R	0.067	R	0.3	R
MAP Strain 1	Milk	0.25	0.633	R	0.067	R	0.3	R
MAP Strain 3	Tissue	0.25	0.633	R	0.067	R	0.3	R
MAP Strain 7	Tissue	0.25	0.633	R	0.067	R	0.3	R
MAP Strain 8B	Blood	0.25	0.633	R	0.067	R	0.3	R
MAP MS 137	Tissue	4	2.532	R	0.268	R	1.2	R
MAP MS 185	Tissue	4	2.532	R	0.268	R	1.2	R
MAP Para 18	ATCC 19698	0.25	0.633	R	0.067	R	0.3	R
MAP Ben	ATCC 43544	0.25	0.633	R	0.067	R	0.3	R
MAP Kay	ATCC C286	0.25	0.633	R	0.067	R	0.3	R
MAP Linda	ATCC 43015	0.25	0.633	R	0.067	R	0.3	R
Non-MAP strains								
*M. avium*	ATCC 25291	4	2.532	R	0.268	R	1.2	R
*M. avium* JF 5	Faeces	4	2.532	R	0.268	R	1.2	R
*M. avium* JF 6	Faeces	4	2.532	R	0.268	R	1.2	R
*M. avium* JF 8	Blood	4	2.532	R	0.268	R	1.2	R
*M. intracellulare* LM1-A	Sputum	4	3.798	R	0.402	R	1.8	R
*M*-*Smegmatis*	ATCC 27199	6	3.798	R	0.402	R	1.8	R
*M. chelonae subsp. chelonea*		>10^a^	6.33	R	0.67	R	3	R
*M. fortuitum subsp. fortuitum*		>10^a^	6.33	R	0.67	R	3	R

*MIC* minimum inhibitory concentration, *CLA* clarithromycin, *CLO* clofazimine, *RIF* rifabutin, *Suscep* susceptibility

^a^MIC was greater than 10 μg/mL and no higher drug concentrations were analyzed

### MAP viability testing

Several culture tubes incubated with MAP and the triple combination of RHB-104 active ingredients that did not show signs of bacterial growth following 6 months of incubation were investigated for drug effects on MAP growth. A volume of 1 mL taken from each culture tube was centrifuged at 13,200 rpm for 5 min and the cell pellet was washed three times with 1X PBS. Each cell pellet was then re-suspended in 800 μL of growth supplement including mycobactin J and inoculated into a new tube of BD Bactec™ MGIT™ TB Medium. The cultures were incubated for 6 months at 37 °C. Tubes were read daily for up to 6 months for signs of growth using BD Bactec™ MGIT™ 320 instrument.

## Results

### Confirmation of MAP identity using IS900 nested PCR

**Fig. 2:**
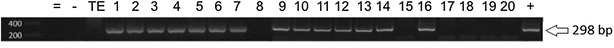
Nested PCR analysis of DNA from representative mycobacterial cultures. *IS900*-based nested PCR (nPCR) was performed on DNA extracts from MAP and other mycobacterial cultures. PCR products were analyzed on 2 % agarose gel and 298 bp amplified fragment is considered positive for MAP (*lane* *20*). *1*: UCF 5, *2*: UCF 8, *3*: MAP 7, *4*: MAP 8B, *5*: MAP Ben, *6*: MAP Kay, *7*: MAP Linda, *8*: MAP Para, *9*: MAP UCF 3, *10*: MAP UCF 4, *11*: MAP UCF 5, *12*: MAP UCF 7, *13*: MAP UCF 8, *14*: *M. avium* JF 7, *15*: *M. avium* JF 8, *16*: MAP Para 18, *17*: *M. avium avium*, *18*: *M. intracellulari* LM1A, *19*: *M. smegmatis*, *20*: *M. tuberculosis*

*IS900* nested PCR was performed on DNA extracts from a total of 35 mycobacterial isolates. The detection of 298 bp on 2 % agarose gel was reported as MAP positive. Overall, 16 cultures were identified as MAP and 19 as other mycobacterial species. Figure 2 shows a representative agarose gel for PCR products of some bacterial cultures.

### Susceptibility of mycobacterial strains to CLA, CLO, RIF, and RHB-104 Active ingredients

**Fig. 3:**
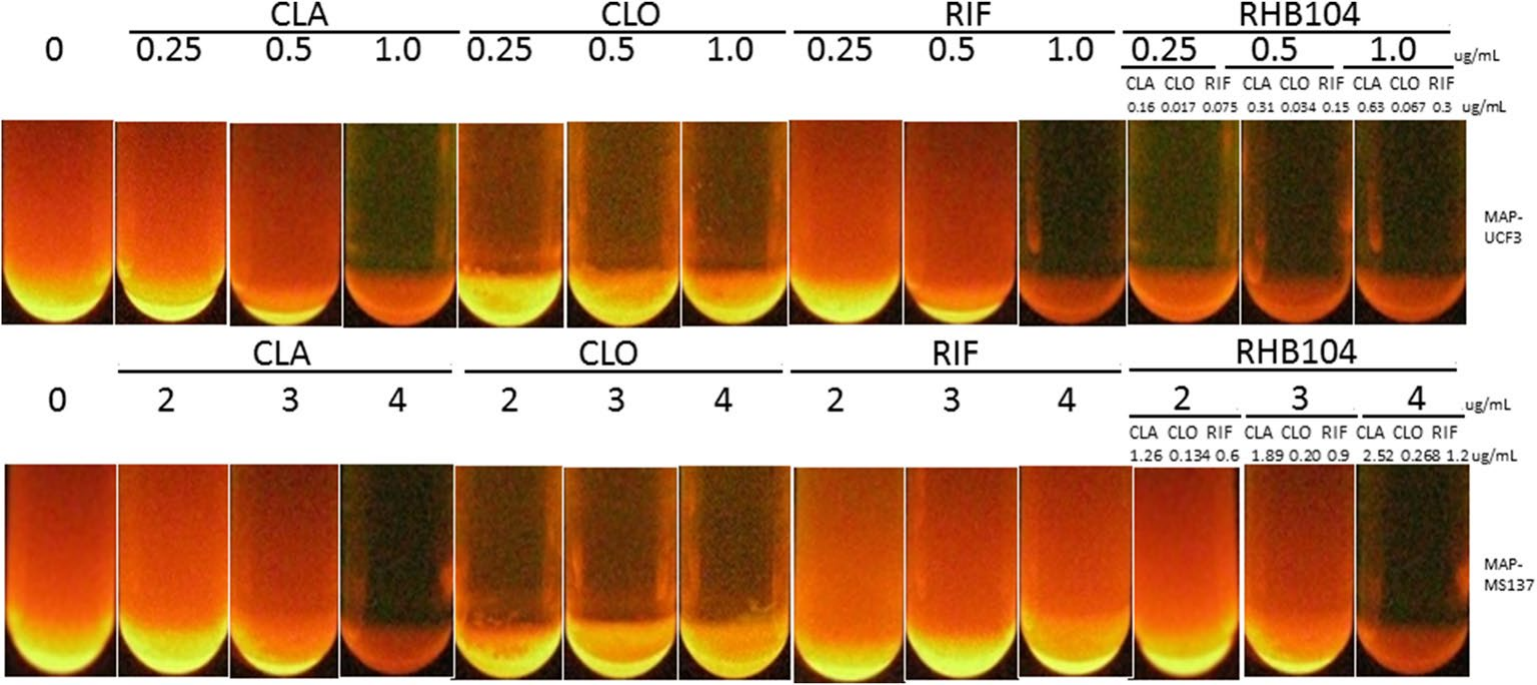
Susceptibility of MAP strains UCF 3 and MS 137 to RHB-104 active ingredients, individually and in triple combination. MGIT para-TB culture media supplemented with 2 μg/mL mycobactin J were inoculated with MAP strains UCF 3 and MAP MS 137. In the presence of CLA, CLO, RIF or RHB-104 at concentrations ranged between 0 and 6 μg/mL. Culture tubes were incubated at 37 °C for a minimum of 10 weeks. Fluorescence in the tube indicated the presence of actively respiring bacteria and an absence of fluorescence indicated lack of bacterial growth

The MIC for CLA, CLO, RIF, and RHB-104 active ingredients against 35 mycobacterial strains are shown in Table 1. The MIC of each drug ranged between 0.25 and 2 μg/mL against 13 out of 16 MAP strains. All drugs had MIC levels between 4 and  >10 μg/mL for the more resistant 3 out of 16 MAP strains, MAP UCF 10, MS 137, and MS 185. Interestingly, MAP MS 137 and MS 185 strains exhibited susceptibility to the triple combination of RHB-104 active ingredients at 4.0 μg/mL (containing 63.3 % CLA—2.53 μg/mL, 6.7 % CLO—0.27 μg/mL, and 30 % RIF—1.2 μg/mL) compared to 4 to >6 μg/mL for each of CLA, CLO and RIF, showing some synergistic activity of the three active ingredients at lower dosages. The MIC of RHB-104 active ingredients for MAP strain UCF 10 was 10 μg/mL representing the most resistant MAP strain in this study. MAP UCF 10 was resistant to CLO and RIF individually at concentrations of >10 μg/mL. Although MAP UCF 10 was susceptible to 10 μg/mL of CLA, it was susceptible to only 6.3 μg/mL when CLA was tested as part of RHB-104 active ingredients solution. Figure 3 shows 2 representative figures of MAP susceptibility to RHB-104 active ingredients, individually and in triple combination.

Among non-MAP strains, the MIC values for all drugs were higher. The reported MIC for CLA, CLO, RIF, and CLA-CLO-RIF among the *M. avium* strains ranged between >1 and  >20 μg/mL. *M. avium* clinical strain JF1 showed high resistance with an MIC for all drugs including CLA-CLO-RIF at >20 μg/mL. Non-MAP strains such as *M. smegmatis*, *M. chelonae, and M. fortuitum* were more resistant than other non-MAP strains *M. scrofulaceum, M. terrae, M. tuberculosis, M. xenopi, and M. vallae* with reported MIC for all drugs ≥10 μg/mL (Table 1).

### Potency of RHB-104

Although Table 1 showed comparable MIC levels of CLA, CLO, RIF, and CLA-CLO-RIF, RHB-104 active ingredients showed more potency against mycobacterial strains. This was determined by further examining each individual drug at their concentrations in the MIC levels of CLA-CLO-RIF (shown in Table 1) against each respective mycobacterial strain. In Table 2, the MIC of CLA–CLO–RIF against 13 MAP strains was 0.25 μg/mL, which comprised 0.158 μg/mL CLA (63.3 % of RHB-104), 0.017 μg/mL CLO (6.7 % of RHB-104), and 0.075 μg/mL RIF (30 % of RHB-104). At these concentrations, MAP showed resistance against each of the 3 individual drugs. In two other MAP strains (MAP MS 137 and MAP MS 185), all individual drugs tested at their concentrations in 4 μg/mL CLA–CLO–RIF were not effective at bacterial inhibition. In Fig. 3, two representative MAP strains incubated with different concentrations of each drug showed the potency of RHB-104 even at low dosages.

Non-MAP strains were more resistant to the drugs used in this study. The MIC of CLA-CLO-RIF against 4 *M. avium* strains was 4.0 μg/mL (Table 2). However, the individual concentrations of CLA, CLO and RIF in 4.0 μg/mL of RHB-104 active ingredients solution were not effective against *M. avium* strains (Table 2). Similar results were observed when other mycobacteria were tested, showing the potency of a the triple combination in RHB-104 active ingredients.

### Synergistic effects in combined drug therapy

**Fig. 4:**
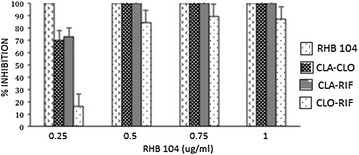
Two-drug combinations showed less potency of bacterial growth inhibition than triple drug combination. A combination of CLA–CLO, CLA–RIF, and CLO–RIF at concentrations of 0.25, 0.5, 0.75, and 1 μg/mL were tested against clinical MAP strain UCF 4. The triple combination of RHB-104 active ingredients showed effective bacterial growth inhibition at a low MIC level of 0.25 μg/mL, whereas the 2-drug combinations showed only partial inhibition

In order to further analyze the synergistic effects of RHB-104 active ingredients CLA–CLO–RIF, 2-drug combinations were tested against MAP UCF 4 at concentrations in RHB-104 ranging from 0.25 to 1.0 μg/mL. As shown in Fig. 4, only the triple combination of CLA–CLO–RIF completely inhibited (100 % inhibition) MAP growth at their concentrations in 0.25 μg/mL RHB-104, whereas MAP growth was partially inhibited by CLA–CLO regimen at 70 % inhibition and CLA–RIF regimen at 15 % inhibition. Moreover, complete inhibition of MAP growth for CLA–CLO and CLA–RIF regimens was only achieved at higher levels of their concentrations in 0.5 μg/mL RHB-104. CLO–RIF regimen was the least effective against MAP, only inhibiting MAP growth by 90 % at their individual concentrations in 1.0 μg/mL of RHB-104.

### Bactericidal effect of RHB-104 active ingredients

Bacterial cell pellet from active culture of MAP UCF 4 incubated with 1 μg/mL CLA-CLO-RIF solution that did not shown signs of growth was washed and re-inoculated into fresh medium without any drugs. Following a 6-month incubation, there was no growth in the culture tube. The experiment was repeated several times with different MAP strains and has resulted in similar outcomes. The effect of the triple combination of the drugs on bacterial cultures could not be reversed. The outcome supports a bactericidal effect for the drug in culture.

## Discussion

Anti-MAP regimens including CLA, RIF, and CLO have been investigated in multiple clinical trials as possible treatments to many diseases including CD cases suspected with MAP implication [24–27]. RHB-104 is a new combination anti-MAP therapy that combines these three unique drugs in a single pill. In this study, we evaluated in vitro the efficacy of RHB-104 active ingredients against clinical MAP strains and other mycobacterial controls.

Initially, we evaluated CLA, CLO, and RIF individually to determine their potency against 16 clinical MAP strains and 19 non-MAP strains. Our data for the MIC of each individual drug against MAP were comparable to previous studies that reported the MIC for CLA in the range between 0.25 and 0.5 μg/mL, CLO at 0.5–1 μg/mL, and RIF at 0.3–0.5 μg/mL [28–31]. Non-MAP strains such as *M. smegmatis*, *M. intracellulare* and *M. avium* strains were susceptible at higher dosages of >4 to 20 μg/mL of CLA, CLO, and RIF which confirmed previous studies [32, 33].

The novelty of this study is focused on the efficacy of RHB-104 active ingredients solution (CLA–CLO–RIF) against 35 microorganisms. This formula has provided anti-MAP growth effect at lower concentrations. The MIC for RHB-104 active ingredients solution against 13 out of 16 MAP strains was 0.25 μg/mL (0.158 μg/mL CLA, 0.017 μg/mL CLO, and 0.075 μg/mL RIF). Certainly, CLA at 0.158 μg/mL, CLO at 0.017 μg/mL and RIF at 0.075 μg/mL when used individually had no measurable effect on MAP growth (Table 2). In fact, we determined the MIC for CLA, CLO and RIF to be 0.25, 0.5, and 0.3 μg/mL, respectively which are multi folds higher than their levels in CLA–CLO–RIF solution. Of course, some MAP strains required higher MIC for each of these drugs (Table 1). Even when these drugs were paired and evaluated against MAP, the anti-MAP growth effect was not significant compared to that of the triple combination. As shown in Fig. 4, at 0.25 μg/mL, which is the MIC for CLA–CLO–RIF against MAP UCF 4, all possible pair combinations of the drugs were no match for the effects of the triple combination which makes the active ingredients in RHB-104. The in vitro anti-MAP growth effect was the most effective when the triple combination was used. The confidence in these data rose from results of several lab trials where CLA, CLO and RIF were tested individually, in pairs and all together forming RHB-104 active ingredients. RHB-104 active ingredients CLA–CLO–RIF also showed potency as a triple combination against non-MAP strains. The MIC for CLA–CLO–RIF solution against these non-MAP strains ranged between 4 and 20 μg/mL. However, when CLA, CLO, and RIF concentrations at the MIC level in CLA–CLO–RIF solution were tested, the bacteria continued to grow, although not very robustly (or partial growth) as the control with no added drugs. Partial growth of mycobacteria in the presence of the drug indicates less potency and in clinical situations. This could be harmful since it may promote acquisition of drug resistance. Overall, our data have shown that lower concentrations of CLA–CLO–RIF solution, which forms the active ingredients of RHB-104 provided more efficacious outcomes in vitro against clinical MAP strains and those microorganisms with higher MIC values.

While CLA appears to be potent in bacterial inhibition at less than 633 or 0.63 μg/mL, it is futile to treat patients with one antibiotic drug for several months because of possible development of antibiotic resistance. CLA is also an inhibitor of CYP34A, an enzyme that is important in drug metabolism [34]. Clarithromycin-mediated inhibition of CYP34A leads to drug-to-drug interactions as well as marked increase of exposure to drugs taken chronically such as simvastatin and pravastatin [35–37]. A combination of antibiotic drugs such those included in RHB-104 can eliminate the problem with drug resistance and exacerbated adverse effects while exhibiting effective anti-MAP activity.

## Conclusion

MAP in humans lack its cell wall [22]; therefore, using antibiotic drugs that target cell wall will not only be inefficient for treatment of CD, but it may also lead to complications by inhibiting some normal flora and rise of multidrug resistance bacteria. The triple combination of CLA, CLO, and RIF in RHB-104 has demonstrated excellent synergistic activity in culture and in inhibition mycobacterial growth. RHB-104 should be considered as the new generation of CLA, CLO and RIF and may be labeled as the new and most effective regimen to treat CD cases associated with MAP infection.

## Abbreviations

5-ASA: 5-aminosalicylates
AIDS: acquired immune deficiency syndrome
CD: Crohn’s disease
CLA: clarithromycin
CLO: clofazimine
DNA: deoxyribonucleic acid
FDA: Food and Drug Administration
GI: gastrointestinal
HIV: human immunodeficiency virus
JD: Johne’s disease
MAB: monoclonal antibodies
MAC: *Mycobacterium avium* complex
MAP: *Mycobacterium avium* subspecies *paratuberculosis*
MGIT: *Mycobacteria* growth indicator tube
MIC: minimum inhibitory concentration
ND: not determined
PCR: polymerase chain reaction
RHB 104: Cocktail antibiotic comprised of clarithromycin, rifabutin, and clofazimine
RIF: rifabutin
RNA: ribonucleic acid
TE: tris-ethylenediaminetetraacetic acid
TNF-α: tumor necrosis factor α
UV: ultraviolet
